# Sex-Specific Sensory Profiles Discriminate Between Sensitization at Twelve Weeks in Patients with Acute Low Back Pain: A Retrospective Study

**DOI:** 10.3390/jcm14020621

**Published:** 2025-01-19

**Authors:** Pieter J. Gräper, Aldo Scafoglieri, Joannes M. Hallegraeff

**Affiliations:** 1 Experimental Anatomy Research Group, Department of Physiotherapy, Human Physiology and Anatomy, Faculty of Physical Education and Physiotherapy, Vrije Universiteit Brussel, Laarbeeklaan 103, 1090 Brussels, Belgium; aldo.scafoglieri@vub.be (A.S.); joannes.marinus.hallegraeff@vub.be (J.M.H.); 2 Department of Master Education, SOMT University of Physiotherapy, Softwareweg 5, 3821 BN Amersfoort, The Netherlands

**Keywords:** sensory modulation, sensory profiles, low back pain, chronicity, Adolescent/Adult Sensory Profile, central nervous system sensitization

## Abstract

**Background/objective:** Low back pain (LBP) is the leading cause of disability worldwide, resulting in enormous socio-economic and personal consequences. Sensory profiles during the acute back pain stage will predict central sensitization symptoms in the chronic pain stage, as central sensitization is the main mechanism behind nociplastic pain and pain chronicity. Therefore, our objective was to establish overall and sex-specific sensory profile cut-off points that distinguish symptoms of central sensitization at 12 weeks, using a retrospective prognostic cohort study design. **Methods:** Two hundred and seventeen patients with acute LBP (<6 weeks) were assessed using Receiver Operator Characteristic analyses. Measurements were taken at baseline using the Adolescent/Adult Sensory Profile and follow-up by the Central Sensitization Inventory at 12 weeks, based on the established Central Sensitization Inventory cut-off points for the overall population at ≥30 and ≥40, female patients at ≥33, and male patients at ≥25. **Results:** In female patients, a Sensory Sensitive cut-off point of ≥30.5 significantly distinguished central sensitization symptoms at 12 weeks, resulting in the following values: Area Under the Curve = 0.81 (95% CI = 0.73; 0.89), sensitivity = 0.89, specificity = 0.63, prevalence = 0.36, positive predictive value = 0.56, negative predictive value = 0.80, and Youden’s index = 0.52. **Conclusions:** The Sensory Sensitive profile distinguishes female patients with acute LBP with and without central sensitization symptoms at 12 weeks. This cut-off point may be useful in identifying individual sensory preferences and addressing maladaptive behavioral responses to sensory stimulation in clinical practice to prevent chronicity.

## 1. Introduction

Acute low back pain (LBP) is the leading cause of disability worldwide, resulting in significant socio-economic consequences and a large increase in prevalence expected by 2050 [1,2].

Acute LBP initiates the development of peripheral sensitization, leading to increased receptiveness to sensory input and the patient’s perceptibility to sensory overstimulation [3,4,5]. Mostly, increased peripheral sensitization is restored to previous levels within a few weeks [6,7,8]. However, when normal tissue-healing time has passed and sensitization does not return to former levels, recovery may be prevented, and acute LBP will transform into chronic LBP after 12 weeks [5,6,8]. This peripheral phenomenon can be considered a bottom-up stressor, which, when combined with top-down psychological stressors, causes sensory discomfort, facilitating sensitization further [6,8]. Several related mechanisms within the peripheral and central nervous system decrease the function of endogenous analgesia, consequently inducing hypersensitivity, which is responsible for central sensitization (CS) and chronic LBP [5,8,9].

Peripheral sensitization and CS, in combination with trait sensory processing characteristics, amplify active (seeking or avoiding sensory stimulation) or passive behavioral responses [10,11]. Trait sensory thresholds and behavioral responses are described as sensory profiles (SPs) in Dunn’s quadrant for sensory processing [3,12,13]. Sensory profiles are a research-based framework assessing sensory preferences and behavioral responses to sensory stimulation in everyday life and are established prognostic factors in the persistence of CS symptoms [3,12,13,14]. In the literature, sex differences are identified in the severity and presentation of CS in chronic musculoskeletal disorders [15,16]. Therefore, in the severity of CS, it is important to account for presented sex differences [15,16].

Although SPs may initiate personalized pain treatment based on individual patient characteristics, such as sex, in the prevention of chronic musculoskeletal pain disorders, cut-off points have not been established yet [11,14]. The cut-off points of SPs are easily applicable in daily clinical practice. They may guide a tailored rehabilitation strategy based on personal sensory preferences and results, thus providing a more patient-centered approach. In addition, maladaptive behavioral responses to sensory stimulation may be the focus of treatment.

Therefore, this study aims to establish sex-based SP cut-off points in patients with acute LBP to identify the risk of CS symptoms in the context of the development of chronic pain.

## 2. Materials and Methods

### 2.1. Study Design

This is a retrospective observational study. Two independent pre-existing data files were merged, measuring CS symptoms and SPs in patients with acute LBP over 12 weeks. As a guideline, the STROBE checklist was used, and this study is reported in adherence with the TRIPOD and REMARK checklists and the SAGER guidelines [17,18,19,20].

### 2.2. Ethics, Settings, and Participants

Data were collected from primary clinical physiotherapy practices where patients reported for consultation, throughout the Netherlands, from January 2016 to March 2017 for data file 1 (ClinicalTrials.gov Identifier: NCT04974229; Medical Ethics Committee (METC) of the University of Groningen, the Netherlands, registration number: M15.169564, approval date 13 February 2015) and from May 2022 to June 2023 for data file 2 (ClinicalTrials.gov Identifier: NCT05097235; Medical Ethics Committee of the University Hospital Brussels (UZB), Belgium, trial number BUN: 1432021000708, approval date 13 April 2022), both according to the declaration of Helsinki (revision 2013) [21].

Pseudo-anonymization was performed according to institutional protocols, after which the cases were consecutively numbered in the merged file. All encoded data were stored in an electronic database in a secure area; therefore, confidentiality and anonymity were guaranteed.

The inclusion criteria were baseline and second measurements after 12 weeks, using the AASP (Adolescent/Adult Sensory Profile) and CSI (Central Sensitization Inventory). All consecutively recruited patients with acute non-specific LBP who were capable of understanding, writing, and reading the Dutch language, with a duration of LBP < 6 weeks, were included. Acute LBP consists of discomfort or pain between the inferior gluteal folds and costal margin, with or without symptoms in the lower limbs, that cannot be attributed to an underlying structural lesion or specific disease (such as a tumor, osteoporosis, fracture, structural deformity, radiculopathy, herniated disc, cauda equina syndrome), without a structural or specific cause explaining the pain, and with or without radiating. Additionally, the patients had an age range of 18–60 years, with a pain-free episode of ≥12 weeks before the current LBP episode [22]. The exclusion criteria were a specific cause of LBP, structural spinal problems (such as pathology of the nervous or vascular system, cancer, or a rheumatic disease, fibromyalgia, previous history of surgery in the lumbar region, or pregnancy), previous spinal operations, and pain that radiates from other parts of the body [22].

### 2.3. Outcome Variable

The relationship between the SPs and the development of chronic pain at twelve weeks was analyzed by determining SP cut-off points in the general LBP population, additionally specified for biological sex (male or female) following the SAGER guidelines [20]. SPs are a research-based framework used to assess sensory processing related to taste/smell, movement, vision, touch, activity levels, and auditive input and evaluate behavioral responses to sensory experiences of everyday life, assessed by the AASP [3,12,13] (Table 1). The AASP identifies SPs by comparing sensory thresholds to normative values in the general population, which are age-based (11–17 years; 18–64 years; ≥65 years) [3,23]. The four SPs, known as Low Registration, Sensation Seeking, Sensory Sensitive, and Sensation Avoiding, are determined based on sensory thresholds combined with adaptive behavioral responses to sensory stimulation [12]. The AASP consists of 60 items, with each sensory profile measured on a subscale of 15 items scored using a Likert scale ranging from 1 to 5, resulting in a minimum score of 15 and a maximum score of 75 [13]. Trait hyper- or hyposensitivity is indicated by a score of ±1 standard deviation (SD) from normative values, reflecting passive or active behavioral responses to sensory stimulation: more, much more, less, or much less than most people [12]. In patients with LBP, the AASP is a valid and reliable measurement tool, with an internal consistency of Cronbach’s alpha of 0.91–0.92 and a test–retest reliability ICC= 0.82–0.87 (95% CI 0.74–0.91) [24]. Construct validity correlates positively and significantly with the following constructs: depression, anxiety (trait and state), helplessness, catastrophizing, rumination, and disability, but Sensation Seeking exhibits a negative correlation [24]. SPs are prognostic factors in predicting CS symptoms at 12 weeks [11,14].

### 2.4. Predictor Variable

CS symptoms can be measured by the CSI questionnaire [16,25]. The CSI highly discriminates between patients with CS symptoms, and patients without CS symptoms [16]. Part A comprises 25 questions on a 5-point Likert scale, each item scoring 0–4 points, resulting in a total score range of 0–100. Part B inventories the presence of 10 diagnosed CS-related syndromes on a binary scale [25]. Commonly, CSI ≥ 40 indicates the presence of CS symptoms, although more recent studies suggest different cut-off points in various populations, conditions, and sexes [16,25,26]. The following factors have excellent internal consistency (Cronbach’s alpha = 0.91): disability and physical symptoms, higher central sensitivity, urological, and dermatological symptoms, and emotional distress [25,27]. When using a general cut-off score of CSI ≥ 30, a female cut-off score of CSI ≥ 33, and a male cut-off score of CSI ≥ 25, the CSI shows high discriminative capabilities between patients with CS symptoms and healthy people and between sexes (χ^2^ test *p* < 0.001–0.008) [16].

### 2.5. Demographic Variables

The following established prognostic variables for chronic LBP were obtained at baseline and follow-up in both data files, after which they were merged and analyzed: age, sex, height, weight, duration of low back pain, pain severity, location of LBP, level of education, occupational demands, and the presence of recurring LBP [27].

### 2.6. Data Analysis

If the missing data for the predictor variables (SPs) in the data files is <0.15, all the cases can be included, and missing values can be considered completely at random [28,29].

Recruitment for the first data file started in 2016, before the COVID-19 pandemic, and that for the second data file started in 2022, after the pandemic. To assess whether COVID-19 influenced pain intensity and sensitivity, statistical analyses were performed on both data files. The recent literature indicates that increased pain intensity or sensitivity, without a clear manifestation of post-COVID-19 pain, is not associated with a previous history of COVID-19 [30]. However, to assess the influence of the pandemic on the obtained data, Receiver Operator Characteristic (ROC) analyses were performed, and SP differences between each data file are ±1SD according to normative values established by Gándara-Gafo (2019) [23].

After obtaining data that showed relevant overlap, they were cleaned, harmonized, and merged, leaving only relevant variables for analysis [31]. Additional baseline characteristics were assessed and reported. After assessing patient characteristics for the general data file and sex differences, ROC analyses were performed, comprising Area Under the Curve (AUC), 95% confidence intervals (CI95%s), cut-off points, sensitivity, specificity, prevalence, and positive and negative predictive values. The Youden’s index was assessed, ranging from zero, with no diagnostic value, to one, indicating a perfect test. Different established cut-off points were analyzed for all cases (CSI ≥ 30 and CSI ≥ 40), for female patients (CSI ≥ 33), and for male participants (CSI ≥ 25) [15,16,25]. IBM SPSS Statistics v. 29.0 was used for statistical analyses.

## 3. Results

**Figure 1:**
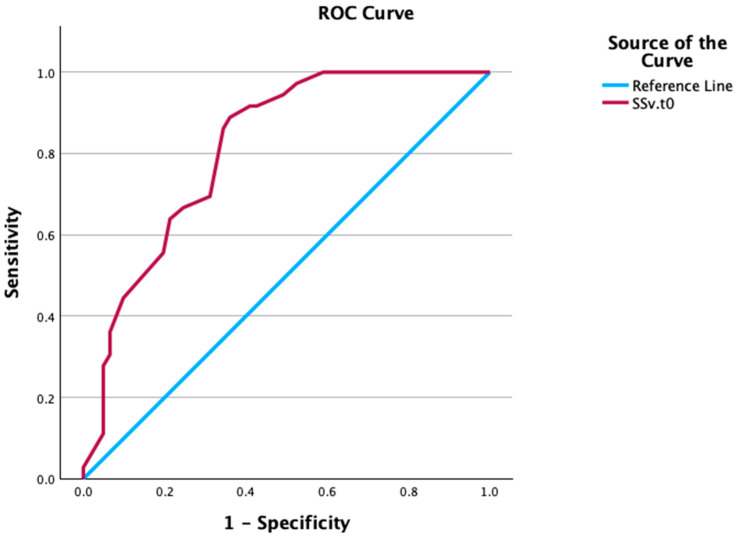
Receiver Operator Curve of the baseline Sensory Sensitive profile in an acute state, and CSI ≥ 33 at 12 weeks in female patients with low back pain.

Both data sets were merged (n = 103 and n = 114 participants), resulting in a total of n = 217 cases. (Table 2 and Table 3). In the variables of interest, missing data were detected at 0.00%, indicating that statistical analyses could be performed without further adjustments [28,31]. In the overall population (CSI ≥ 30 or CSI ≥ 40) and male population (CSI ≥ 25), SPs do not discriminate between the presence of CS symptoms at 12 weeks, except for the Sensory Sensitive profile in the female population (CSI ≥ 33). In the female population, SPs discriminate between the presence of CS symptoms across all presented cut-off points in the literature, both in the overall chronic pain population (CSI ≥ 30 or CSI ≥ 40) and in the sex-specific cut-off points (CSI ≥ 25 and CSI ≥ 33) [16,25] (Table 4, Table 5 and Table 6 and Figure 1).

Based on the ROC analyses performed on each data file separately, the cut-off points differ well within 1 SD between both data files from the normative values according to Gándara-Gafo (2019) [23]. However, the original sample sizes may separately not be large enough to support robust statistical analyses.

## 4. Discussion

The results of this study show that a low sensory threshold and a passive behavioral response to sensory input are predictive of CS development and may suggest that actively regulating sensory input to prevent sensory discomfort can be considered to prevent chronic pain in the acute pain stage [32,33]. The novelty of this study is the identification of cut-off points for SPs in the development of CS symptoms at 12 weeks in patients with acute LBP, suggesting differentiation between female and male patients [15,16]. SPs in the acute LBP stage may contribute to the prevention of chronic LBP, as sensory preferences and behavioral responses lead to a more patient-centered approach.

When assessing commonly used CSI cut-off points in the general musculoskeletal chronic pain population (CSI ≥ 30 and CSI ≥ 40), a differentiation is established in sex-specific cut-off points (male: CSI ≥ 25; female: CSI ≥ 33), which is confirmed in this study [15,16,25]. In line with previous research, our results show that Sensory Sensitive has the strongest capability in discriminating between CS symptoms at 12 weeks, and the discriminatory power increases when a higher cut-off point is chosen from CSI ≥ 30, CSI ≥ 33, or CSI ≥ 40 [11,14]. A sex-specific cut-off point of CSI ≥ 33 in female patients is established, which provides a significant distinction between patients with CS symptoms and those without CS symptoms, corresponding to a cut-off point for the Sensory Sensitive score of ≥30.5 [16]. While other CSI cut-off points are suggested and their discriminatory power is suitable for clinical practice, they are not specified by sex [16].

To establish a genuine effect on CSI scores after 12 weeks, baseline CSI scores should not be used as an independent prognostic factor because peripheral sensitization is already present and is a normal response in acute LBP [6,34]. Peripheral sensitization can be considered an effect modifier or a confounding factor, rather than an independent prognostic factor, resulting in pain experiences for at least 3 months to assume CS [9,35].

No correlation was found between pain severity and CS symptoms, consistent with findings from previous studies [11,14,16,24].

In line with previous studies, no association was found between baseline CS symptoms, pain experiences, and SPs, suggesting that pain experiences and pain severity are not identical to CS symptoms [11,14,16,24]. As a result, it can be hypothesized that CS is less influenced by synaptic hypersensitivity and can be considered an adaption of neural modulation and cognitive processes of the central nervous system. As SPs and the CSI are not associated with pain severity or pain ‘experiences’, the association with the clinical relevance of quantitative sensory testing is ambiguous [36].

According to Dunn (1997), SPs are stable trait characteristics over 12 weeks [12]. In contrast, pain and CSI scores differ significantly at 12 weeks (as sensitization in the acute phase indicates peripheral sensitization, and CS can only be assumed after ≥3 months when LBP is at a chronic phase) [3,6,12].

### 4.1. Clinical Implications

The result of our study shows that sex-specific cut-off points should be considered when assessing SPs in acute LBP treatment.

The AASP assesses all four sensory profiles (Low Registration, Sensation Seeking, Sensory Sensitive, and Sensation Avoiding) according to Dunn’s quadrant of sensory processing, resulting in a score for each SP on a continuous scale, each with different predictive values and cut-off points [12].

By using CSI ≥ 33, both Sensation Avoiding and Sensory Sensitive can be used in clinical practice for female patients. Although the discriminatory power of Sensory Sensitive is more favorable in contrast to CSI ≥ 40, the discriminatory power of Sensation Avoiding is more favorable in CSI ≥ 40. However, CSI sex-specific cut-off points are available, making it appropriate to use CSI ≥ 33 for female patients [16]. Additionally, Sensory Sensitive and Sensation Avoiding measure different constructs (higher and lower sensory thresholds) and cannot be combined [13]. Therefore, it seems apparent that different rehabilitation interventions should be considered to address sensory discomfort. As they measure different constructs, i.e., Sensory Sensitive is associated with a passive behavioral response and Sensation Avoiding is associated with an active avoidance response, it seems apparent that different rehabilitation interventions should be considered to address sensory discomfort. However, it can be hypothesized that the sole use of the Sensory Sensitive cut-off point to distinguish central sensitization at 12 weeks in female patients may be sufficient, dismissing the Sensation Avoiding outcome. When Sensory Sensitive, containing 15 patient-reported outcome measure items, is enough to distinguish CS symptoms at 12 weeks, this may be beneficial for patients and clinicians as it is less time-consuming [13].

The clinical benefits consist of the early detection of patients who potentially develop chronic LBP in the acute stage by identifying patients’ sensory thresholds and behaviors to sensory stimulation [12]. In line with recent guidelines, interventions in the acute phase are suggested, including efforts to avoid sensory discomfort and adjust maladaptive behaviors, as sensory profiles predict CS symptoms in the acute stage [11,14,22,32,37]. A low sensory threshold combined with passive behavioral responses to sensory stimulation is most prone to the development of CS symptoms, and, therefore, chronic pain conditions. Sensory Sensitive is associated with a low sensory threshold and passive behavior to avoid sensory stimulation, both of which increase sensory discomfort [33]. To adjust passive maladaptive behaviors, cognitive behavioral therapy principles and neuroscience education are suggested to adjust top-down cognitive processes and minimize sensory discomfort [11,33]. Bottom-up sensory discomfort and stress resulting from sensory overstimulation can be minimized by adaptive behaviors, such as avoiding stimulation, which may resolve peripheral sensitization [14,33].

In clinical practice, cognitive behavioral interventions in the acute phase may prevent maladaptive behaviors and may result in fewer symptoms of CS after 12 weeks.

### 4.2. Limitations

The sample size distribution between the different established cut-off points on the CSI outcome score shows that CSI ≥ 40 is not sufficient for either male (n = 9) or female patients (n = 18) or for the overall population (n = 27).

Previously established cut-off points have been used to identify CS as a dichotomous outcome, including statistical uncertainty [16,25]. Therefore, SP cut-off points should be considered relative to the suggested CSI cut-off points.

Lacking a gold standard method to identify CS, the presence of symptoms of CS can objectively be evaluated using biomarkers containing brain imaging, such as assessing reduced gray matter volume, alterations in resting state functional connectivity, and lab results, such as altered levels of brain-derived neurotrophic factor (BDNF) and gamma-aminobutyric acid (GABA) [38,39,40,41]. Semi-objectively, CS can be assessed by quantitative sensory testing, where objective sensory stimulation is subjectively evaluated by the patient. However, these measurements are time-consuming and highly burdensome for the patient, and additional training is required [16,42,43]. According to Neblett et al. (2024), quantitative sensory testing modalities including conditioned pain modulation, temporal summation, pressure pain threshold, heat pain threshold, and cold pain threshold correlate significantly with the CSI [5]. The CSI, a Patient Reported Outcome Measure, is recommended to quantify symptoms of CS in clinical practice and highly discriminates between patients with and without CS symptoms. However, it is not clear whether it measures nociceptive sensitization or psychological constructs related to CS [5,16,43].

A limitation may be that the sample distribution between a previous episode of LBP during a patient’s lifetime (52.5%) and a first episode of LBP (47.0%) is relatively even. Therefore, chronic pain processes may already be present in some patients, even though all participants had an absence of LBP for >12 weeks before inclusion. As such, the LBP episode is diagnosed as acute LBP [22].

Another limitation may be that the first data file was obtained before the COVID-19 pandemic, whereas the second data file was obtained during the pandemic, which may have biased the results. However, a previous history of COVID-19 is not associated with increased pain intensity or sensitivity without a clear manifestation of post-COVID-19 pain [30]. Due to the retrospective character of this study, patients with post-COVID pain may have been included during the recruitment of the second data file.

Participation bias may be present in this study, as the psychological nature of using PROMs in research as a reason to decline participation may likely be present in a group with more prominent psychological problems.

In addition, unknown confounding factors influencing CS development are always present, but not factored.

### 4.3. Recommendations

To better understand the predictive role of Sensory Sensitive and Sensation Avoiding in clinical practice on CS symptom development, a predictor of treatment effect study should be conducted. Investigating behavioral interventions in acute LBP and its effect on developing chronic pain may further widen our understanding of LBP.

## 5. Conclusions

Sex differences are present when discriminating between CS symptoms at 12 weeks by a low sensory threshold (Sensory Sensitive and Sensation Avoiding). A passive behavioral response (Sensory Sensitive) to sensory stimulation increases discriminative capabilities further. Therefore, Sensory Sensitive, with a cut-off point ≥ 30.5, is recommended to discriminate between the presence of symptoms of central sensitization at 12 weeks in female patients with acute low back pain. Female patients with a low sensory threshold may benefit from interventions on passive behavioral responses to sensory stimulation and may reduce CS symptoms at 12 weeks.

## Figures and Tables

**Table 1:** Sensory profile quadrants: relating neurological sensory thresholds to behavioral responses. Adapted from Dunn (1997) [12].

		Self-Regulating Behavior by Sensory Preferences	
		*Passive*	*Active*
**Sensory Threshold**	*High*	Low Registration	Sensation Seeking
	*Low*	Sensory Sensitive	Sensation Avoiding

**Table 2:** Continuous baseline demographics of acute low back pain patients (n = 217).

Variables									
	Total Sample n = 217	CSI < 30 n = 134	CSI ≥ 30 n = 47	CSI < 40 n = 180	CSI ≥ 40 n = 27	CSI < 33 n = 60	CSI ≥ 33 n = 36	CSI < 25 n = 68	CSI ≥ 25 n = 43
Age (yrs.) mean (SD)	42 (12.1)	42 (12.0)	43 (12.1)	42 (12.0)	41 (13.1)	42 (11.6)	44 (13.2)	42 (12.1)	43 (11.5)
Height (m.) mean (SD)	1.78 (0.1)	1.78 (0.1)	1.8 (0.1)	1.78 (0.1)	1.8 (0.1)	1.70 (0.1)	1.70 (0.1)	1.84 (0.1)	1.84 (0.1)
Weight (kg.) mean (SD)	81.3 (15.7)	81.4 (15.8)	81.5 (16.2)	81.3 (15.8)	82.2 (16.6)	72.4 (11.8)	76.9 (16.5)	87.9 (14.0)	87.4 (13.1)
Duration (wks.) Mean (SD)	3.0 (1.8)	3.0 (1.9)	2.9 (1.5)	2.9 (1.9)	3.1 (1.4)	3.1 (2.0)	2.8 (1.6)	2.9 (1.7)	2.7 (1.3)
NPRS mean (SD)	6.2 (1.9)	6.1 (1.9)	6.4 (1.8)	6.3 (1.8)	6.0 (1.9)	6.3 (2.0)	6.5 (2.0)	6.1 (1.7)	6.5 (1.3)

Abbreviations: CSI = Central Sensitization Inventory; n = number of participants; SD = standard deviation.

**Table 3:** Categorical baseline demographics of acute low back pain patients (n = 217).

Variables									
	Total Sample n (%)	CSI < 30 n (%)	CSI ≥ 30 n (%)	CSI < 40 n (%)	CSI ≥ 40 n (%)	CSI < 33 n (%)	CSI ≥ 33 n (%)	CSI < 25 n (%)	CSI ≥ 25 n (%)
Sex									
Female	102 (47)	54 (53)	43 (42)	79 (78)	18 (18)	61 (60)	36 (53)	44 (43)	53 (52)
Male	115 (53)	80 (70)	31 (27)	102 (89)	9 (8)	92 (80)	19 (17)	68 (59)	43 (37)
Location									
Lower back	166 (76)	106 (79)	53 (71)	140 (77)	19 (70)	49 (80)	28 (78)	53 (78)	29 (67)
Leg	50 (23)	28 (20)	20 (27)	41 (22)	7 (26)	12 (20)	7 (19)	15 (22)	14 (33)
Education									
Lower	32 (14)	20 (14)	11 (14)	30 (16)	1 (4)	7 (12)	6 (17)	12 (18)	6 (14)
Middle	104 (47)	69 (51)	32 (43)	89 (49)	12 (44)	28 (46)	12 (33)	37 (54)	24 (56)
Higher	81 (37)	45 (33)	31 (41)	62 (34)	14 (52)	26 (43)	18 (50)	19 (28)	13 (30)
Work									
Sedentary	100 (46)	59 (50)	37 (50)	81 (44)	15 (56)	32 (53)	16 (44)	26 (38)	22 (51)
Mostly sedentary	22 (10)	15 (11)	5 (6)	17 (9)	3 (11)	4 (7)	4 (11)	10 (15)	2 (45)
Standing– walking	69 (31)	46 (34)	21 (28)	63 (34)	4 (15)	22 (36)	12 (33)	22 (32)	11 (26)
Physical heavy	23 (10)	13 (9)	9 (12)	18 (9)	4 (15)	3 (5)	2 (6)	9 (13)	8 (19)
Recurrent LBP									
Yes	114 (53)	68 (51)	40 (54)	91 (50)	17 (63)	28 (46)	19 (53)	36 (53)	25 (58)
No	102 (47)	66 (49)	33 (45)	90 (50)	9 (33)	33 (54)	16 (44)	32 (47)	18 (42)

Abbreviations: CSI = Central Sensitization Inventory; n = number of participants.

**Table 4:** Sensory profile cut-off point assessment predicting central sensitization symptoms of the **total sample** of participants (n = 217).

**CSI ≥ 30**	**AUC (CI95%)**	**Cut-Off Point**	**Sens.**	**Spec.**	**Prev.**	**PPV**	**NPV**	**Youden’s Index**
*LR*	0.69 (0.62; 0.76)	25.5	0.76	0.53	0.34	0.28	0.89	0.29
*SSk*	0.57 (0.49; 0.65)	44.5	0.61	0.58	0.34	0.00	0.99	0.19
*SSv*	0.75 (0.68; 082)	31.5	0.70	0.68	0.34	0.38	0.90	0.39
*SA*	0.72 (0.65; 0.79)	33.5	0.54	0.77	0.34	0.42	0.86	0.31
**CSI ≥ 40**	**AUC (CI95%)**	**Cut-off point**	**Sens.**	**Spec.**	**Prev.**	**PPV**	**NPV**	**Youden’s Index**
*LR*	0.65 (0.54; 0.75)	26.5	0.74	0.52	0.13	0.00	0.99	0.26
*SSk*	0.56 (0.44; 0.68)	43.5	0.70	0.47	0.12	0.00	1.00	0.17
*SSv*	0.76 (0.66; 0.86)	33.5	0.67	0.77	0.12	0.04	0.98	0.43
*SA*	0.74 (0.63; 0.84	36.5	0.59	0.82	0.12	0.04	0.99	0.41

Abbreviations: CSI = Central Sensitization Inventory at 12 weeks; AUC = Area Under the Curve; CI95% = 95% confidence interval; Sens.= sensitivity; Spec. = specificity; Prev. = prevalence; PPV = positive predictive value; NPV = negative predictive value; LR = baseline Low Registration sensory profile; SSk = baseline Sensory Seeking sensory profile at baseline; SSv = baseline Sensation Sensitive sensory profile; SA = baseline Sensory Avoiding sensory profile.

**Table 5:** Male patient sensory profile cut-off point CSI ≥ 25 assessment predicting central sensitization symptoms at 12 weeks, and general CSI ≥ 30 and CSI ≥ 40 cut-off points for male patients (n = 115).

**CSI ≥ 25**	**AUC (CI95%)**	**Cut-Off Point**	**Sens.**	**Spec.**	**Prev.**	**PPV**	**NPV**	**Youden’s** **Index**
*LR*	0.56 (0.45; 0.66)	25.5	0.63	0.53	0.37	0.00	N/A	0.16
*SSk*	0.54 (0.43; 0.66)	43.5	0.58	0.57	0.37	0.00	N/A	0.16
*SSv*	0.71 (0.61; 0.81)	29.5	0.77	0.66	0.37	0.26	0.90	0.43
*SA*	0.68 (0.58; 0.78)	26.5	0.84	0.50	0.37	0.33	0.84	0.34
**CSI ≥ 30**	**AUC (CI95%)**	**Cut-off point**	**Sens.**	**Spec.**	**Prev.**	**PPV**	**NPV**	**Youden’s** **index**
*LR*	0.63 (0.52; 0.74)	25.5	0.71	0.54	0.27	0.03	N/A	0.25
*SSk*	0.56 (0.43; 0.68)	49.5	0.36	0.79	0.27	0.00	N/A	0.14
*SSv*	0.67 (0.55; 0.78)	29.5	0.71	0.58	0.27	0.06	0.96	0.29
*SA*	0.65 (0.54; 0.77)	26.5	0.84	0.45	0.27	0.03	0.98	0.29
**CSI ≥ 40**	**AUC (CI95%)**	**Cut-off point**	**Sens.**	**Spec.**	**Prev.**	**PPV**	**NPV**	**Youden’s** **Index**
*LR*	0.42 (0.27; 0.58)	19.5	0.89	0.19	0.08	0.00	N/A	0.08
*SSk*	0.51 (0.30; 0.72)	53.5	0.22	0.90	008	0.00	N/A	0.12
*SSv*	0.61 (0.40; 0.82)	34.5	0.44	0.86	008	0.00	N/A	0.31
*SA*	0.54 (0.36; 0.73)	30.5	0.67	0.57	0.08	0.00	N/A	0.24

Abbreviations: CSI = Central Sensitization Inventory at 12 weeks; AUC = Area Under the Curve; CI95% = 95% confidence interval; Sens. = sensitivity; Spec. = specificity; Prev. = prevalence; PPV = positive predictive value; NPV = negative predictive value; LR = baseline Low Registration sensory profile; SSk = baseline Sensory Seeking sensory profile at baseline; SSv = baseline Sensation Sensitive sensory profile; SA = baseline Sensory Avoiding sensory profile.

**Table 6:** Female patient sensory profile cut-off point CSI ≥ 33 assessment predicting central sensitization symptoms at 12 weeks, and general CSI ≥ 30 and CSI ≥ 40 cut-off points for female patients (n = 102).

**CSI ≥ 30**	**AUC (CI95%)**	**Cut-Off Point**	**Sens.**	**Spec.**	**Prev.**	**PPV**	**NPV**	**Youden’s** **Index**
*LR*	0.72 (0.62; 0.82)	23.5	0.88	0.47	0.43	0.53	0.72	0.36
*SSk*	0.55 (0.43; 0.67)	44.5	0.70	0.49	0.42	0.23	0.81	0.19
*SSv*	0.80 (0.71; 0.89)	28.5	0.88	0.60	0.42	0.63	0.81	0.49
*SA*	0.77 (0.70; 0.86)	36.5	0.56	0.87	0.42	0.60	0.74	0.43
**CSI ≥ 33**	**AUC (CI95%)**	**Cut-off point**	**Sens.**	**Spec.**	**Prev.**	**PPV**	**NPV**	**Youden’s** **Index**
*LR*	0.73 (0.63; 0.83)	23.5	0.92	0.45	0.36	0.44	0.87	0.37
*SSk*	0.55 (0.43; 0.67)	39.5	0.92	0.22	0.36	0.00	0.98	0.13
*SSv*	0.81 (0.73; 0.89)	30.5	0.89	0.63	0.36	0.56	0.80	0.52
*SA*	0.76 (0.66; 0.86)	36.5	0.58	0.83	0.36	0.58	0.84	0.42
**CSI ≥ 40**	**AUC (CI95%)**	**Cut-off point**	**Sens.**	**Spec.**	**Prev.**	**PPV**	**NPV**	**Youden’s** **Index**
*LR*	0.73 (0.62; 0.84)	26.5	0.94	0.50	0.18	0.11	1.00	0.44
*SSk*	0.55 (0.42; 0.69)	42.5	0.83	0.35	0.18	0.00	1.00	0.18
*SSv*	0.81 (0.71; 0.90)	30.5	1.00	0.54	0.18	0.39	0.94	0.54
*SA*	0.82 (0.71; 0.93)	36.5	0.83	0.80	0.18	0.11	0.94	0.63

Abbreviations: CSI= Central Sensitization Inventory at 12 weeks; AUC = Area Under the Curve; CI95% = 95% confidence interval; Sens.= sensitivity; Spec. = specificity; Prev.= prevalence; PPV = positive predictive value; NPV = negative predictive value; LR = baseline Low Registration sensory profile; SSk = baseline Sensory Seeking sensory profile at baseline; SSv = baseline Sensation Sensitive sensory profile; SA = baseline Sensory Avoiding sensory profile.

## Data Availability

Data are unavailable due to privacy restrictions.
